# Validity of the short version of the Upper Limb Functional Index with 10 items in Brazilian patients with chronic musculoskeletal dysfunction in the upper limb

**DOI:** 10.1590/1806-9282.20242013

**Published:** 2025-08-08

**Authors:** Daniel Santos Rocha, Henrique Yuji Takahasi, Cid André Fidelis de Paula Gomes, Almir Vieira Dibai-Filho

**Affiliations:** 1 Universidade Federal do Maranhão, Postgraduate Program in Physical Education – São Luís (MA), Brazil.; 2 Sarah Network of Rehabilitation Hospitals – São Luís (MA), Brazil.; 3 Universidade Nove de Julho, Postgraduate Program in Rehabilitation Sciences – São Paulo (SP), Brazil.

**Keywords:** Upper limb, Factor analysis, Functioning, Reported outcome measures

## Abstract

**OBJECTIVE:**

The aim of this study was to validate the structural and criterion validity of the short version of the Upper Limb Functional Index with 10 items in Brazilian patients with musculoskeletal dysfunction in the upper limb.

**METHODS:**

The structure of the Upper Limb Functional Index with 10 items and with one domain was tested using confirmatory factor analysis with model fit evaluated using comparative fit index, Tucker-Lewis index, root mean square error of approximation, standardized root mean square residual, and chi-square/degree of freedom. Criterion validity was assessed using Spearman's correlation coefficient (rho) to correlate the Brazilian versions of Upper Limb Functional Index with 25 items and Upper Limb Functional Index with 10 items.

**RESULTS:**

We included 150 patients, mostly women, with an average age of 52.21±12.09 years, diagnosed with chronic musculoskeletal dysfunction in the upper limbs. The Upper Limb Functional Indexwith 10 items showed sufficient fit indices (CFI=0.970, TLI=0.962, RMSEA=0.054, SRMR=0.078, chi-square/DF=1.43). Criterion validity showed an excellent correlation between Upper Limb Functional Indexwith 25 items and Upper Limb Functional Index with 10 items, with rho=0.900 (p<0.001).

**CONCLUSION:**

The Upper Limb Functional Index with 10 items demonstrated sufficient unidimensional structure and high correlation with the original version. We recommend the use of Upper Limb Functional Index with 10 items for assessing upper limb functioning in Brazilian patients with chronic musculoskeletal dysfunction.

## INTRODUCTION

The clinical evaluation of functioning of the upper limb is commonly conducted using patient-reported outcome measures (PROMs). Among the commonly used instruments, the Upper Limb Functional Index (ULFI) stands out for its valid internal structure, appropriate construct, and acceptable reliability^1,2^. This instrument demonstrated sufficient clinimetric properties in the version adapted for Brazil^3^, as well as for other cultures and languages, such as Turkish^4^, Spanish^5^, French-Canadian^6^, Italian^7^, Arabic^8^, Urdu^9^_,_ and Persian^10^.

Historically, the ULFI was originally created by Gabel et al.^1^ in 2006 in Australia, in parallel with two other instruments that have similar structures, i.e., each consisting of 25 items and three response options (yes, partly, or no)^2^. These instruments are: the Spine Functional Index (SFI), specific for the evaluation of spinal function^11^, and the Lower Limb Functional Index (LLFI), specific for the evaluation of lower limb function^12^. Of these three instruments, the SFI and LLFI have shortened versions, consisting of 10 items, as reported in recent publications^13–15^.

In this context, several studies have presented short versions of PROMs by excluding items to reduce application time, decrease the possibility of errors in completion, and minimize the probability of unanswered items, without compromising the quality of the collected information^16–20^. Regarding the upper limbs, the short version of the Disabilities of the Arm, Shoulder, and Hand Questionnaire (DASH)^21^, called QuickDASH, is an example of questionnaire reduction with wide clinical application^22^. However, some studies point to the debatable one-dimensional internal structure of QuickDASH^2,23^.

In this context, the ULFI with 25 items stands out as an instrument that specifically assesses a region of the body, being easy to understand and applicable to various musculoskeletal disorders^3^. Therefore, considering the benefits of short instruments^16^, the ULFI with 10 items (ULFI-10) proposal contributes to more coherent and efficient evaluation initiatives, corroborating the reduced versions of SFI-10 and LLFI-10^13–15^.

Given the above and emphasizing the importance of reducing scales and questionnaires, the present study aims to validate the structural and criterion validity of the short version of the ULFI with 10 items (ULFI-10) in Brazilian patients with musculoskeletal dysfunction in the upper limb. The hypothesis of our study is that the ULFI-10 presents a valid unidimensional structure and is sufficiently correlated with the long version of the instrument.

## METHODS

### Study design and ethical aspects

This is a prospective cross-sectional study. We performed secondary analyses of partial data from a previously published study^3^. Data were collected at the Sarah Network of Rehabilitation Hospitals (São Luís, Maranhão, Northeast Brazil). All participants provided written consent. This study was approved by the institution's Research Ethics Committee (opinion number 2.990.249) and conducted according to the Declaration of Helsinki.

### Participants

The appropriate sample size was calculated following recommendations by the Consensus-based Standards for the selection of health Measurement INstruments (COSMIN): seven times the number of items in the questionnaire, provided the sample size is ≥100 participants^24^. Inclusion criteria were individuals of both sexes, over 18 years old, Brazilian Portuguese speakers, with musculoskeletal pain and/or dysfunction in the upper limbs lasting ≥12 weeks (chronic dysfunction), with a defined orthopedic diagnosis. Patients with a history of surgery less than 6 months prior, infectious diseases, central neurological conditions that compromise the functioning of the upper limb (such as stroke, Parkinson's disease, spinal cord injury), any type of cancer, and severe psychiatric disorders preventing questionnaire completion were excluded.

### Upper Limb Functional Index with 10 items

ULFI-10 is a specific questionnaire that investigates the function in the proximal, central, and distal regions of the upper limb. The instrument comprises 10 items with three response options: yes (1 point), partly (0.5 points), and no (0 points). The result is obtained by summing the marked points and multiplying by 10. The product result is subtracted from 100, resulting in a final score ranging from 0 to 100. Higher scores indicate better functioning. The ULFI-10 is a reduction of the original 25-item version (ULFI-25), suggested by the instrument's creator (Dr. Charles Philip Gabel). The 10 retained items are: items 3, 6, 10, 11, 12, 13, 17, 20, 23, and 24.

The Brazilian version of the ULFI with 25 items presents sufficient measurement properties, with unidimensionality, excellent reliability, and internal consistency. Furthermore, the construct is valid when correlated with the QuickDASH, the 36-Item Short Form Health Survey (SF-36), and the Numerical Rating Pain Scale (NRPS)^3^.

### Statistical analysis

Descriptive statistical analysis was performed, presenting mean and standard deviation values for quantitative variables and using absolute numbers and percentages for qualitative variables. Descriptive analysis was conducted using SPSS software (version 17.0, Chicago, IL, USA).

Confirmatory factor analysis (CFA) was performed using R Studio (Boston, MA, USA) with the lavaan and semPlot packages. CFA was conducted using a polychoric matrix and the robust diagonally weighted least squares (RDWLS) extraction method, as recommended for ordinal data^25,26^. The following model fit indices were considered: comparative fit index (CFI), Tucker-Lewis index (TLI), root mean square error of approximation (RMSEA) with 90% confidence interval (CI), standardized root mean square residual (SRMR), and chi-square/degree of freedom (DF). Values >0.90 were considered sufficient for CFI and TLI, while values <0.08 were considered sufficient for RMSEA and SRMR. Values <3.00 were considered sufficient for interpreting chi-square/DF^27,28^. Factor loadings ≥0.40 were considered sufficient for each item^16,29,^.

Criterion validity was assessed using Spearman's correlation coefficient (rho) to correlate the Brazilian versions of ULFI-25 and ULFI-10. Criterion validity was achieved when rho was ≥0.70^30^.

## RESULTS

Initially, the study had 160 participants, but 10 patients were excluded for not completely filling out the ULFI items. Thus, the study included 150 patients with medical diagnoses of chronic musculoskeletal pain and/or dysfunction in the upper limbs. Most of the sample were women, married, with an average age of 52.21±12.09 years (age range: 22–90 years), with incomplete or complete high school education. The main upper limb dysfunctions presented by patients were subacromial impingement syndrome (34%), carpal tunnel syndrome (26%), epicondylalgia (11.33%), trigger finger (6%), and hand osteoarthritis (6%). Table 1 describes the sociodemographic and clinical characteristics of the study participants.

**Table 1:** Sample characteristics (n=150).

Variables		Mean (standard deviation) or number (%)
	Age (years)	52.21 (12.09)
	Sex (female)	128 (85.3)
Marital status		
	Single	45 (30)
	Married	78 (52)
	Divorced	16 (10.7)
	Widower	11 (7.3)
Schooling		
	Elementary (incomplete or complete)	24 (16)
	High school (incomplete or complete)	67 (44.7)
	University education (incomplete or complete)	35 (23.3)
	Postgraduate (incomplete or complete)	24 (16)
Affected side		
	Right	46 (30.7)
	Left	29 (19.3)
	Bilateral	75 (50)
Dysfunction		
	Subacromial impingement syndrome	51 (34)
	Carpal tunnel syndrome	39 (26)
	Epicondylalgia	17 (11.33)
	Trigger finger	9 (6)
	Hand osteoarthritis	9 (6)
	De Quervain tenosynovitis	5 (3.33)
	Bicipital tendonitis	5 (3.33)
	Calcific tendonitis	5 (3.33)
	Adhesive capsulitis	2 (1.33)
	Other	8 (5.33)
ULFI		
	25 items (score, 0–100)	54.51 (21.10)
	10 items (score, 0–100)	56.33 (21.90)

ULFI: Upper Limb Functional Index.

In the CFA, the fit indices were sufficient for ULFI-10 (chi-square/DF <3, CFI and TLI >0.90, RMSEA and SRMR <0.08), as shown in Table 2. This table also describes the ULFI-10 items and their respective factor loadings (≥0.40). Regarding criterion validity, we observed a correlation magnitude of 0.900 (p<0.001) between the ULFI-25 and ULFI-10 versions, demonstrating the maintenance of evaluative capability even after reducing the number of items.

**Table 2:** Factor loadings and fit indices of Upper Limb Functional Index with 10 items (n=150).

Item description	Factor loadings
1. I avoid heavy jobs, e.g., cleaning, lifting more than 5 kg or 10 lb, gardening, etc.	0.62
2. I have the pain/problem almost all the time.	0.55
3. I have difficulty with normal home or family duties and chores.	0.68
4. I sleep less well.	0.57
5. I need assistance with personal care, e.g., washing and hygiene.	0.70
6. My regular daily activities (work, social contact) are affected.	0.61
7. I have difficulty putting my arm into a shirt sleeve or need assistance dressing.	0.69
8. I have difficulty eating and/or using utensils (e.g., knife, fork, spoon, chopsticks).	0.75
9. I use the other arm more often.	0.51
10. I have difficulty with buttons, keys, coins, taps/faucets, containers, or screw-top lids.	0.71
Fit indices	
Chi-square/DF	1.43
CFI	0.970
TLI	0.962
RMSEA (90%CI)	0.054 (0.009, 0.086)
SRMR	0.078

DF: degree of freedom; CFI: comparative fit index; TLI: Tucker-Lewis index; RMSEA: root mean square error of approximation; CI: confidence interval; SRMR: standardized root mean squared residual. Sufficient fit indices (chi-square/DF <3, CFI and TLI >0.90, RMSEA and SRMR <0.08).

The Brazilian Portuguese version of ULFI-10 can be freely accessed at questionariosbrasil.blogspot.com.

## DISCUSSION

This study confirmed the unidimensionality of the ULFI-10, meaning all 10 items are sufficiently explained by the same latent variable (functioning). This finding corroborates the unidimensional structure found in the Brazilian ULFI-25 version^3^, as well as the Spanish^5^ and original versions^1^. Conversely, the Turkish^4^, Urdu^9^, and Italian^31^ versions found a two-dimensional structure.

When comparing the Brazilian versions of the ULFI-25^3^ and the ULFI-10, we identified that the reduced version (ULFI-10) showed better values for all the presented fit indices (CFI=0.970, TLI=0.962, RMSEA=0.054, SRMR=0.078, chi-square/DF=1.43) than the 25-item version (CFI=0.918, TLI=0.910, RMSEA=0.063, SRMR not reported, chi-square/DF=1.75). However, both versions (with 10 and 25 items) demonstrated a valid internal structure, supported by factor analysis.

Considering the Brazilian version of the SFI-10, an instrument with a similar structure to the ULFI-10, but aimed at evaluating spinal function, we observed results similar to those of the present study. That is, the internal structure with 10 items of the SFI showed more sufficient fit indices (CFI=0.959, TLI=0.947, RMSEA=0.068, chi-square/DF=1.88) than the 25-item SFI version (CFI=0.896, TLI=0.887, RMSEA=0.070, chi-square/DF=1.94)^14^.

From a clinical point of view, the ULFI with 25 items presents adequate clinimetric properties, which support its use in patients with upper limb dysfunctions^3^. However, based on our results, we recommend using the short version (ULFI-10) because it maintains the same evaluative capacity and is quicker to complete. This recommendation is mainly based on the high magnitude of correlation between the short and long versions, a value higher than the acceptability cutoff for criterion validity (>0.70)^24^.

The present study has limitations. The recommendation for using ULFI-10 is based on structural and criterion validity. However, it is important for future studies to evaluate other measurement properties, such as reliability and construct validity. The sample studied consisted of patients with chronic musculoskeletal dysfunctions. Other dysfunctions related to the upper limbs should be addressed in future studies, such as acute musculoskeletal dysfunctions and those caused by central nervous system alterations or oncological processes. The sample of our study was collected in a rehabilitation hospital; therefore, patients from primary health care or physiotherapy clinics may have different response patterns, and this factor is another important point to be considered in the present study. Finally, our sample was composed mostly of women (85.3%). The explanatory factor for this higher proportion of women in the sample is complex, but the scientific literature is clear in establishing that women seek health services more than men^32^.

## CONCLUSION

The ULFI-10 demonstrated sufficient internal structure and excellent correlation with the original version. Therefore, we recommend using this measurement instrument in assessing the functioning of the upper limbs in Brazilian patients with chronic musculoskeletal dysfunctions.

## Data Availability

The datasets generated and/or analyzed during the current study are available from the corresponding author upon reasonable request.
